# Family Medicine Implementation to Maternal and Child Health in Rural Japan: A Narrative Review

**DOI:** 10.7759/cureus.60305

**Published:** 2024-05-14

**Authors:** Ryuichi Ohta, Chiaki Sano

**Affiliations:** 1 Community Care, Unnan City Hospital, Unnan, JPN; 2 Community Medicine Management, Shimane University Faculty of Medicine, Izumo, JPN

**Keywords:** general medicine, community health services, health manpower, family practice, rural health, child health services, maternal health services

## Abstract

This research provides a critical narrative review of maternal and child health (MCH) in rural Japan, reflecting broader challenges faced by aging societies globally. The study explores the intertwined roles of professional and lay care in sustaining rural communities, emphasizing the unique position of family medicine and primary care in enhancing MCH services. The scarcity of healthcare resources, particularly the shortage of obstetricians and the weakening of traditional community support systems, underscores the challenges in these areas. Our review method involved a comprehensive search of PubMed for articles published from April 2000 to August 2024, focusing on MCH issues in rural Japan. This study highlights several critical gaps in rural MCH provision: the migration of medical professionals to urban centers, the transformation of social structures affecting traditional caregiving, and the lack of specialized MCH training among primary care physicians. We discuss potential solutions such as incentivizing obstetric care in rural areas, integrating MCH education within family medicine curricula, and revitalizing community-based support systems. By addressing these issues, the research aims to formulate actionable strategies to bolster MCH services, thus ensuring better health outcomes and sustainability of rural communities in Japan and similar settings worldwide.

## Introduction and background

In the fabric of thriving communities, the health and well-being of mothers and children are fundamental elements that ensure continuity and sustainability [1]. The significance of maternal and child health (MCH) extends beyond immediate health outcomes, influencing communities' very survival and development [2]. This is particularly crucial in aging societies, where a diminishing population poses a severe challenge to maintaining the vitality and administration of community systems [3]. Enhanced MCH not only supports individuals' physical and emotional health but also fosters environments conducive to retaining and attracting young generations [4]. Such care underpins the sustainability of communities, ensuring they remain vibrant and livable spaces for all residents.

Despite its importance, the provision of MCH is fraught with challenges, especially in rural areas where there often needs to be more healthcare professionals and resources [5]. This scarcity is compounded by the migration of medical professionals to urban centers, leaving rural populations underserved [4]. Moreover, traditional lay care systems, which have historically played a vital role in community-based support, are weakening due to evolving social structures and relationships within these communities [6].

Recognizing the critical role of family medicine and primary care, this research focuses on these fields as pivotal to enhancing rural MCH [7]. With their broad expertise and community-oriented approach, these medical specialties are uniquely positioned to bridge professional and lay care, strengthening the overall care framework [8]. This study aims to perform a narrative review to delineate current trends and challenges in rural MCH in Japan, a nation emblematic of global aging trends. Through this review, we seek to identify gaps in current practices and propose actionable solutions that could serve as a blueprint for revitalizing rural health systems in Japan and similar contexts globally. Thus, this academic research aims to critically analyze the state of rural MCH in Japan, highlight the interplay between professional and lay care, and suggest practical strategies for improvement through narrative review.

## Review

We performed a narrative review regarding rural MCH in rural contexts using PubMed. We searched PubMed for original articles regarding the issues in rural MCH from April 2000 to August 2024. Regarding articles written in English, our search strategy was based on the following title/abstract keywords: (((Maternal) or ((Child) or (Pediatric)) AND (Care)) AND (rural) AND (Japan)). The reference lists of relevant studies were also reviewed to identify research that might also need to be included. Reading the included articles, we summarize the issues of MCH in rural contexts and discuss possible action plans for the sustainability of rural MCH.

Lack of obstetricians in rural areas

One of the critical issues is the need for obstetricians in rural areas. In Japan, the number of obstetricians has increased gradually, but rural areas need more physicians because most obstetricians work in urban areas. Statistics show that the number of rural obstetricians was 926 in 1996, further decreasing by 25% in 2016. Because of the decrease in physicians, pregnant women visit urban clinics and obstetrics hospitals [9]. The percentage of pregnant women transported to cities from rural places recently increased significantly. The reason for the transfer was mainly from threatened preterm labor [10]. In our case, working at a rural community hospital, there are two obstetricians, but they live in urban areas. Thus, rural physicians call them when pregnant patients need emergent care. Stabilization of pregnancy is vital in rural contexts for safe pregnancy and delivery.

Social and cultural capital for childbearing

In rural contexts, nuclear families have increased, and childbearing has lost social and cultural capital. In 2018, the rate of nuclear families was 76.5%, continually decreasing, so parents cannot get help from their parents in childbearing [11]. In addition, human relationships in rural contexts have weakened recently because of privacy issues. Rural couples cannot get social support from communities effectively compared to the past. In the past, childbearing could be supported by communities.

By participating in community activities, children can acquire appropriate table manners reflecting cultural values, socializing, family bonding, and food appreciation through cultural capital such as preserving traditional dietary habits, eating rules, and intergenerational commensality. Promoting social and cultural capital can improve healthy practices for nutrition education programs [12].

Relationship between primary care physicians and children

In Japan, primary care physicians are not used to dealing with MCH because middle-aged to older primary care physicians do not have official MCH education [13]. Japanese medical education has specialized in specialist education for 30 years [14]. Most community primary care physicians were previously specialists and converted to family medicine or general practice [15]. Thus, they have a sense of negativity about MCH. Although Japan educated more family physicians in 10 years and provided multiple educational curricula for specialists about MCH, there are middle-aged to older primary care physicians in rural contexts who do not care for maternal and pediatric patients. In rural communities, such primary care physicians do not construct effective relationships with mothers and children in their communities, respecting continuity of care [13].

Losing interest in vaccination among children

Vaccination is vital for MCH to prevent critical diseases such as pneumonia and meningitis. However, young adults tend to avoid vaccination because of the misinformation on social media about its harmful effects and side effects. They are affected by social media, becoming vaccine phobia, fearing the vaccination is inappropriate. Significantly, the number of vaccinations against COVID-19 and influenza is decreasing, so more maternal and pediatric patients come to the hospitals because of these diseases, impinging on their lives [16]. Avoiding other vaccines, such as pneumonia and influenza, can increase vaccine-preventable diseases (VPDs). A previous study showed that coordination and community orientation can increase influenza vaccine uptake [17]. Respecting patient experience and the involvement of community members should be improved for better vaccine uptake.

Lack of education regarding maternal and child health in primary care

Japanese family physicians may lack education about MCH. In Japan, physicians' specialization has advanced, and obstetricians and pediatricians mainly perform maternal and child care [14]. Thus, in urban areas, family physicians primarily care for older patients and can consult patients needing maternal care of obstetricians [18]. However, in rural contexts, because of the lack of obstetricians and pediatricians, family physicians need to deal with MCH. Rural family physicians in Japan need more training opportunities for MCH to compensate for the scarcity of consultations by obstetricians. Developing maternity care training programs for family physicians can enable them to engage in MCH in rural areas [19].

Benefit of improved maternal and child health in rural Japan for women, children, and families

Increased Access to Obstetric Care

Addressing the shortage of rural obstetricians would greatly benefit pregnant women traveling to urban areas for care. This improvement can reduce the risks associated with delayed or inadequate prenatal care, such as complications in pregnancy and childbirth [20]. Quality improvement could involve incentives for obstetricians to practice in rural areas, telemedicine, and training more local healthcare workers in obstetrics. Concretely, the number of midwives can be increased for effective maternal care in rural medical institutions because the increase in the number of physicians needs more time than midwives.

Education and Support in Maternal Health

Enhanced primary care education about MCH can empower women with the knowledge and skills to better manage their health and pregnancies, leading to healthier outcomes for both mother and child. For the practical education of women, rural medical professionals should be motivated more about MCH to sustain rural communities and improve the younger generation's motivation for having children [19].

Improved Early Life Health Outcomes

Better prenatal and postnatal care directly impacts children's health, reducing the incidence of preventable diseases and developmental issues. This could be achieved through regular checkups, vaccinations, and growth monitoring. In rural contexts, more primary care physicians should be motivated to provide children with vaccinations and regular health checks and enhance the continuity of care in family medicine [21].

Vaccination Awareness and Uptake

Addressing misinformation and vaccine hesitancy through education and community outreach can increase vaccine uptake, protecting children from preventable diseases. Misinformation and fake news on social media can affect rural young adults' perception of vaccinations [16]. In-person communication and community approaches in rural contexts should drive continual education about the effectiveness of vaccination.

Strengthened Family Health Literacy

Educating families about MCH, nutrition, and child development can strengthen their overall health literacy, enabling them to make informed decisions about their health. MCH has been advanced, such as the advancement of vaccination and effective maternal care. Still, middle-aged to older people may not acknowledge such knowledge, especially in rural contexts, because of the lack of knowledge updates [22].

Support Systems

Reviving and strengthening community support systems can provide families with social and cultural capital to raise children in a healthy environment [23]. This involves promoting community-based programs focusing on traditional dietary practices, child-rearing, and family bonding. Although, owing to respect for privacy in rural Japan, the young generation may avoid accepting help from others in communities, a systems approach to MCH respecting social and cultural bonds should be promoted.

Health System Development

Integrating MCH into primary care, especially in rural areas, can lead to a more comprehensive and responsive health system [24]. Developing a more robust primary care system that includes MCH can ensure continuity of care and better health outcomes. To effectively implement MCH into primary care in rural medicine in Japan, rural physicians and citizens should be motivated to learn the primary care system for the sustainability of rural medicine.

Quality Improvement

Applying quality improvement principles such as process optimization, staff training, and resource allocation can enhance MCH services' efficiency and effectiveness. This involves continuous monitoring and evaluation of MCH programs to identify areas for improvement. Reconsidering the role of family physicians should be essential, especially in rural contexts [24]. Transmission of some of the roles of obstetricians and pediatricians to family physicians should be driven for the sustainability of rural medicine.

Building Resilience in Health Systems

Strengthening MCH in rural areas contributes to the health system's overall resilience, enabling it to better respond to various challenges, including demographic changes and epidemics. The number of family physicians in rural areas has increased gradually, so for resilience in rural healthcare, the functions of family physicians in MCH should be facilitated and expanded [19].

Community Engagement and Education

Enhancing community awareness about MCH, including the importance of vaccinations and traditional health practices, can foster a more health-conscious and supportive community. By applying quality improvement methods to address specific challenges, such as the shortage of rural obstetricians, the weakening of social and cultural support systems, and issues around vaccination and primary care education, we can create a more robust, efficient, and effective health system that better serves the needs of all its members [19].

Discussion

Table 1 shows the evaluation of the process and outcomes in this action plan.

**Table 1:** Interventions and outcomes in each theme MCH: maternal and child health

Themes	Process	Outcome
Enhance rural obstetric care	Financial incentives, housing solutions, educational opportunities, collaboration with university hospitals, implementation of telemedicine, training in obstetrics and prenatal care	Increase the number of obstetricians, increase the usage of MCH care, increase the number of other professionals engaged in MCH care
Strengthen primary care education in MCH	Revising the family medicine curriculum, continuing education programs	Increase the confidence of family physicians in MCH care, increase the number of family physicians engaging in MCH care
Foster community-based support systems	Promoting traditional child-rearing and dietary practices, addressing vaccination awareness and uptake, public education campaigns, establishing more accessible vaccination services	Improve the nutritional conditions of children, increase the rate of vaccination among children
Quality improvement in health services	Mentorship programs for newer physicians, support systems for healthcare workers' wellness, involving community members, developing robust emergency response plans, collaboration among healthcare providers, community leaders, and other stakeholders	Retention of new physicians, retention of other medical professionals, increase in the number of community members in MCH care, reduction in transfer time from primary care to tertiary care, enabling ongoing monitoring and evaluation

Enhance Rural Obstetric Care

A comprehensive strategy that integrates various key initiatives is essential to effectively enhance rural obstetric care [24]. Addressing the shortage of obstetricians in rural areas is paramount, requiring programs that offer financial incentives, housing solutions, and educational opportunities to attract and retain these vital healthcare professionals [25]. Collaborating with rural university hospitals can facilitate the deployment of obstetrics and gynecology department members to rural hospitals, significantly improving the availability of specialized care.

Telemedicine has emerged as a crucial tool in bridging the gap in healthcare provision, especially during the COVID-19 pandemic, which has accelerated its adoption. In rural Japan, telemedicine has proven effective in providing expert consultations and follow-up care. It demonstrates its potential to significantly impact MCH by enabling access to specialized medical advice and care remotely [26].

The role of local health workers, including nurses and midwives, cannot be overstated. Enhancing their obstetrics and prenatal care training is crucial for improving immediate and accessible care in rural areas. Japan's part-time approach of integrating retired nurses and midwives into the rural MCH care system is a model of how existing human resources can effectively address healthcare gaps [19].

Strengthen Primary Care Education in MCH

Strengthening primary care education focusing on MCH is another critical aspect of the strategy. The revision of the family medicine curriculum to incorporate MCH modules aims to better prepare residents for the unique challenges of rural healthcare [27]. This includes developing practical skills and patient-centered approaches essential for effective MCH care. Continuing education programs for current family physicians in rural areas, offering workshops and online courses, can further enhance their knowledge and skills, thereby improving patient- and family-oriented care. This educational approach is complemented by fostering interdisciplinary collaboration among healthcare providers, essential for delivering comprehensive care that spans the continuum of maternal and child health needs [21].

Foster Community-Based Support Systems

Community-based support systems are pivotal in reinforcing cultural and social capital in rural areas. Promoting traditional child-rearing and dietary practices through community activities and strengthening familial and societal bonds are essential for the sustainability of rural communities [28]. Moreover, addressing vaccination awareness and uptake is critical. Public education campaigns that counter misinformation and highlight the importance of vaccinations in community health can significantly improve public health outcomes. Establishing more accessible vaccination services through mobile clinics and community health centers is essential for increasing vaccination rates and enhancing overall community health [16].

Quality Improvement in Health Services

Quality improvement in health services is a continuous process that must be guided by patient feedback and health outcomes. Implementing a system for professional development, including mentorship programs for newer physicians and support systems for healthcare workers' wellness, addresses the need to prevent burnout and promote job satisfaction [29]. This also ensures that ethical practice is maintained across all MCH initiatives, respecting patient autonomy and cultural values.

The involvement of community members in health planning and decision-making processes fosters a sense of ownership and responsibility, enhancing the impact of MCH initiatives. Furthermore, developing robust emergency response plans tailored to rural settings is critical for ensuring preparedness and resilience in adverse weather and geographical challenges [30]. Effective collaboration among healthcare providers, community leaders, and other stakeholders is crucial for the regular collection and analysis of data. This enables ongoing monitoring and evaluation of MCH initiatives, ensuring that services can be continuously adapted and improved based on community feedback.

## Conclusions

Enhancing rural obstetric care requires a holistic approach that addresses the multifaceted challenges of rural healthcare provision. Through targeted strategies aimed at improving healthcare professional recruitment and retention, leveraging telemedicine, enhancing primary care education, fostering community-based support systems, and ensuring continuous quality improvement, this comprehensive strategy aims to significantly improve the accessibility and quality of MCH services in rural areas. This approach not only addresses the immediate healthcare needs but also ensures the sustainability and resilience of rural healthcare systems.
